# Effects of Omitting Non-confounding Predictors From General Relative-Risk Models for Binary Outcomes

**DOI:** 10.2188/jea.JE20170226

**Published:** 2019-03-05

**Authors:** John Cologne, Kyoji Furukawa, Eric J. Grant, Robert D. Abbott

**Affiliations:** 1 Department of Statistics, Radiation Effects Research Foundation, Hiroshima, Japan; 2 Department of Epidemiology, Radiation Effects Research Foundation, Hiroshima, Japan; 3 Center for Epidemiologic Research in Asia, Shiga University of Medical Science, Shiga, Japan

**Keywords:** bias, binary outcomes, general relative-risk model, generalized nonlinear model, logistic regression, omitted covariate

## Abstract

**Background:**

The effects, in terms of bias and precision, of omitting non-confounding predictive covariates from generalized linear models have been well studied, and it is known that such omission results in attenuation bias but increased precision with logistic regression. However, many epidemiologic risk analyses utilize alternative models that are not based on a linear predictor, and the effect of omitting non-confounding predictive covariates from such models has not been characterized.

**Methods:**

We employed simulation to study the effects on risk estimation of omitting non-confounding predictive covariates from an excess relative risk (ERR) model and a general additive-multiplicative relative-risk mixture model for binary outcome data in a case-control setting. We also compared the results to the effects with ordinary logistic regression.

**Results:**

For these commonly employed alternative relative-risk models, the bias was similar to that with logistic regression when the risk was small. More generally, the bias and standard error of the risk-parameter estimates demonstrated patterns that are similar to those with logistic regression, but with greater magnitude depending on the true value of the risk. The magnitude of bias and standard error had little relation to study size or underlying disease prevalence.

**Conclusions:**

Prior conclusions regarding omitted covariates in logistic regression models can be qualitatively applied to the ERR and the general additive-multiplicative relative-risk mixture model without substantial change. Quantitatively, however, these alternative models may have slightly greater omitted-covariate bias, depending on the magnitude of the true risk being estimated.

## INTRODUCTION

Binary regression models are often used to estimate the association between an exposure and disease risk with adjustment for other covariates. Adjustment covariates can include confounders, as well as risk factors not associated with exposure (non-confounding predictors). It is well known that omitting confounders can result in bias; what concerns us here is omitting non-confounding predictors. With ordinary linear regression, it does not matter—in terms of bias—whether or not we adjust for additional covariates not associated with included covariates, but such adjustment can increase precision for estimating the effects of the covariates of interest because of reduced residual variation.^1^ This “conventional wisdom” does not apply, however, with generalized linear models (GLM), except with the identity link and log link functions,^2^^–^^4^ and this issue has received renewed attention recently in terms of case-control studies of genetic risk factors.^5^^,^^6^ Estimates of GLM parameters of included covariates can change with omission of covariates associated with outcome but not associated with included covariates (“omitted-covariate bias”); the change is towards the null value of no risk (attenuation) with certain classes of link functions, including logistic regression.^1^^,^^3^ Omitting covariates from a GLM can also effect asymptotic efficiency—also depending on the class of link function—with efficiency gains occurring in the case of the logistic link.^7^

The published work to date assumes a linear predictor. In many applications, it is desirable to perform the analysis using alternative, or “general”, relative-risk (RR) models,^8^^–^^10^ specifically the linear relative risk (or excess relative risk [ERR]) model: ERR = RR − 1,^11^^,^^12^ where RR is the relative risk (we assume that RR ≥1 for exposure, so that ERR ≥0). The effect of omitted covariates does not appear to have been assessed with these more general models. Therefore, we assessed omitted-covariate bias with the ERR model and with a more general relative-risk mixture model that combines components for additive and multiplicative effects of exposure on the RR.

## METHODS

### Mathematical details

We are interested in general relative-risk models of the form

$$\text{logit}(p)=\text{log}\left(\frac{p}{1-p}\right)=\sum_{i=0}^{s}\alpha_{i}Z_{i}+R_{\beta }(X),$$

where p=Pr(Y=1|Z,X) for binary outcome *Y* (coded 1 if the outcome of interest is observed and 0 otherwise); ***Z*** is a vector of adjustment covariates (*Z*_0_ is 1 for the intercept); and *X* is a risk factor (exposure) of interest, which is non-negative, can be discrete or continuous, and has risk expressed through some function *R*_β_(*X*) depending on unknown parameter(s) β. We do not deal here with interactions or joint effects of multiple risk factors because our interest is in the effect of including or omitting other factors (elements of ***Z***) on the risk for a single exposure of interest. This form of model is called “general” because it generalizes the ordinary logistic regression model where *R*(*X*) = β_L_*X* (the subscript “L” signifying ordinary logistic regression). The odds ratio from case-control data approximates the risk ratio (relative risk), and β_L_ from ordinary logistic regression is the log odds ratio corresponding to a one unit difference in risk factor *X*. General relative-risk risk models include a wide range of dose-response shapes and joint effects of multiple factors because they are not constrained to be linear on the scale of the link function (the logit link in the case of logistic regression).^13^

Despite the generality of *R*_β_(*X*), we focus on the ERR model because it is one of the most commonly used forms of general relative-risk model. The ERR model is defined by *R*_β_(*X*) = log(1 + β_E_*X*); we refer to β_E_ as the “ERR parameter”. An important distinction between the ERR model and the ordinary logistic regression model concerns interpretation of the effect of a unit change in the risk factor. With the logistic model, a one-unit increase in the value of the risk factor results in a relative (multiplicative) change $e^{\beta_{\text{L}}}$ in the RR (ie, RR*_X_*_+1_ = $e^{\beta_{\text{L}}}$ RR*_X_*), whereas, with the ERR model, a one-unit increase in the value of the risk factor results in an additive change β_E_ in the RR (ie, RR*_X_*_+1_ = RR*_X_* + β_E_). Therefore, we can speak of the ERR as a risk “per unit exposure”, but we cannot speak of the RR as a risk per unit exposure.

General relative-risk models can also be used to assess departure from strictly additive or strictly multiplicative models for the RR. The log odds mixture model proposed by Thomas^8^ has

$$R_{\beta_{\text{M}}}(X)=\lambda \beta_{\text{M}}X+(1-\lambda )\text{log}(1+\beta_{\text{M}}X).$$ (1)

The power model of Breslow and Storer^9^ has

$$R_{\beta_{\text{P}}}(X)=\{\begin{array}{ll} \frac{{\left(1+\beta_{\text{P}}X\right)}^{\lambda }-1}{\lambda } & \lambda \neq \text{0}\\ \text{log}(1+\beta_{\text{P}}X) & \lambda \text{=0} \end{array}.$$

With both families of models, the ERR model arises when λ = 0, which is a convenient starting point for testing non-additivity of joint effects of multiple risk factors. We also recommend the paper by Breslow and Storer as a generally readable motivation and explanation of the ERR model.

The ERR model can be written as

$$\frac{p}{1-p}=e^{\alpha \prime Z}(1+\beta_{\text{E}}X)$$ (2)

on the odds scale, where ***α*** and ***Z*** are vectors. Strictly speaking, model (2) is a model for the excess odds ratio, but it is generally referred to as an ERR model given the usual odds ratio approximation to the RR that applies with a rare outcome. Indeed, with nested case-control data, one actually estimates the RR, rather than the odds ratio, when fitting a binary regression model.^14^ Therefore, in this paper, we will use “odds ratio” and “relative risk” interchangeably.

As an example, consider the nested case-control study of radiation, endogenous hormones, and breast cancer in female atomic-bomb survivors, based on 57 post-menopausal breast cancer cases and 109 non-cases matched on age and counter-matched on radiation dose.^15^^,^^16^ Although the purpose of that study was to estimate the interaction between radiation and endogenous estradiol, as well as to assess potential mediation by estradiol of the radiation risk, we ignore those two aspects here and focus on the radiation risk alone merely for illustration. Two other covariates, body mass index (BMI; kg/m^2^) and number of full-term pregnancies (parity), are known risk factors (larger parity being protective), so the question “should they be adjusted?” arises. Crude RRs for these covariates were 1.12 (95% confidence interval [CI], 1.01–1.25) for a one-unit increase in BMI and 0.71 (95% CI, 0.55–0.90) for an increase of 1 in parity. With both covariates centered at their mean values among controls (23.0 kg/m^2^ for BMI and 3.0 live births for parity) and with parity treated as a quantitative variable, Table 1 shows that the estimated ERR for radiation decreases when BMI, parity, or both are omitted. The standard error and CI width also decrease (precision increases) with either or both covariates omitted. *P* values are generally the same or larger with omitted covariates, although omission of parity alone results in a slightly reduced *P* value.

**Table 1.** Effect of omitting covariates on estimated ERR in the Radiation and Breast Cancer Study

Covariates omitted	Estimated ERR	SE	95% likelihood bounds	Likelihood ratio *P* value
Adjusted for parity and BMI	1.21	0.89	0.36, 3.71	0.012
BMI omitted	1.09	0.82	0.13, 3.27	0.014
Parity omitted	1.08	0.79	0.15, 3.16	0.011
BMI and parity omitted	0.91	0.70	0.10, 2.67	0.017

BMI, body mass index; ERR, excess relative risk; SE, standard error.

The two adjustment variables (BMI and parity) are not considered potential confounders,^17^^,^^18^ so results in Table 1 are qualitatively similar to what would be expected from omitting non-confounding covariates with ordinary logistic regression. However, because the ERR model is not linear on the scale of any link function, it is not a GLM and results pertaining to bias and precision with a GLM cannot be assumed to apply. Therefore, it is important to assess how bias and precision of the estimated parameters that quantify the risk for exposure are affected by omitting covariates in the case of general relative-risk models, such as the ERR model.

The ERR model (2) can also be written

$$\text{logit}(p)=\alpha \prime Z+\text{ln}(\text{1}+\beta_{\text{E}}X).$$ (3)

If any elements of ***Z*** are omitted, the possibly mis-specified ERR is $\beta_{\text{E}}^{*}$ in

$$\text{logit}(p^{*})=\alpha^{*}\prime Z^{*}+\text{ln}(1+\beta_{\text{E}}^{*}X)$$ (4)

where the asterisk ‘^*^’ represents the situation with one or more elements of ***Z*** omitted. Neither model (3) nor model (4) is a GLM, but either may be fit with the generalized nonlinear model (GNM) package gnm^19^ in R (R Foundation for Statistical Computing, Vienna, Austria) or with the PECAN or GMBO modules in Epicure (Risk Sciences International, Ottawa, Ontario).^13^ The mixture model (1) can also be fit with these programs. R code to specify these models is provided in eMaterial 1 (see “**R code for the generalized nonlinear models**”). We explored the bias and precision of the estimate ${\hat{\beta }}_{\text{E}}^{*}$ of $\beta_{\text{E}}^{*}$ using simulation, as described in the next section.

### Simulation study

We conducted a simulation study to examine whether the omitted-covariate bias and precision with the general relative-risk models depart substantially from the bias and precision results with GLMs. We generated data, including independent effects of the adjustment covariates under either the ERR model, the logistic model, or the additive-multiplicative RR mixture model, and analyzed the data using the correct model (the same model as was used to generate the data) with or without the other covariates adjusted in the analysis. We examined the bias and precision with the mixture model with an intermediate value of the mixture parameter (λ = 0.5), in addition to the mixture-parameter values corresponding to the logistic model (λ = 1.0) and the ERR model (λ = 0.0).

Data on *X* and *Z* for the simulation were generated to mimic the exposure and two covariates—one continuous and one discrete (ordinal)—in the nested case-control study of radiation exposure and breast cancer described above, with a wide range of risk parameters for each covariate (not based solely on the actual study results). Let *X* be radiation dose and ***Z*** be a vector containing two elements: a continuous log-normally distributed variable transformed by taking the logarithm after dividing by the mean, and a discrete variable with possible values 0, 1, 2, …, or 8 (for simplicity we will call these two variables *BMI* and *parity*, but the simulation results can be generalized to any similar continuous and ordered categorical covariates). For each hypothetical cohort member, random values of exposure and the two covariates were generated by independently sampling from their distributions in the cohort, as reflected in the nested case-control study. Radiation doses were randomly drawn from an exponential distribution with mean 0.35 using the R function rexp. BMI values were randomly sampled from a log-normal distribution, with mean 3.11 and standard deviation 0.15 on the log scale, using the R function rlnorm. Parity was randomly selected from a multinomial distribution with probabilities {0.076, 0.069, 0.179, 0.235, 0.169, 0.132, 0.066, 0.047, 0.027} for values {0,1, …, 8}.

Case prevalence at the reference levels of covariates (prevalence among non-exposed subjects with BMI and parity at their mean values), *p*_0_, was set at 0.015, 0.05, or 0.1. After the exposure and two covariates were randomly drawn for each member of the hypothetical cohort, the probability of being a case (*p*) was calculated according to the true risk model under study (whichever one of ERR, logistic, or mixture), with the selected risk parameter for each variable: RR 1.0, 1.25, 1.5, or 1.75 for BMI; RR 1.0, 0.9, 0.75, or 0.5 for parity; and ERR 0.1, 0.25, 0.5, or 1.0 for radiation (corresponding to RR 1.1, 1.25, 1.5, or 2.0 for radiation, respectively). Each hypothetical subject was then assigned an outcome *Y* (case [1] or control [0]) using a random Bernoulli draw (R function rbinom) given that subject’s calculated value of *p*. All cases and an unmatched sample of controls were then selected from the simulated cohort; two simulated cohort sizes (*N*) were used: 5,000 and 20,000. Two control:case sampling ratios (*m*) were studied: 2:1 and 5:1. A 5:1 ratio is assumed to be sufficient for achieving close to full-cohort efficiency in matched studies,^20^ whereas a 2:1 ratio is not efficient with nested case-control sampling.^21^ With large case prevalence and large effects of covariates, the 5:1 ratio can result in a case-control sample size that exceeds the cohort size; such configurations were excluded from the simulation study. BMI and parity were centered in fitted models, as in the actual study (described above), and parity was incorporated as a quantitative covariate. A total of 2,000 simulations was run under each configuration; results from duplicated runs differed by <1.5%. Simulations were conducted in R for Windows version 3.1.3. To facilitate comparing bias with the ERR model to that with logistic regression, we converted the logistic regression estimate to an excess RR as follows: exponentiate the logistic-regression estimate ${\hat{\beta }}_{\text{L}}$ and subtract 1 ($e^{{\hat{\beta }}_{\text{L}}}-1$), then calculate the difference from the true relative risk minus 1 ($e^{\beta_{\text{L}}}-1$) — ie, the bias is $(e^{{\hat{\beta }}_{\text{L}}}-1)-(e^{\beta_{\text{L}}}-1)=e^{{\hat{\beta }}_{\text{L}}}-e^{\beta_{\text{L}}}$.

## RESULTS

Depending on sample size (more often with small samples and low disease prevalence) and true ERR β_E_ (especially with β_E_ close to zero), simulated case-control samples occasionally arose in which, by chance, the mean value of exposure among controls was larger than that among cases, resulting in a negative estimate of ${\hat{\beta }}_{\text{E}}$. This alone is not problematic, but sometimes there arose during the iterations a negative value of the estimate of ${\hat{\beta }}_{\text{E}}$, such that ${\hat{\beta }}_{\text{E}}X\leq -1$ for some hypothetical cohort member’s generated dose value (this tended to occur more often when effects of the other covariates were small); the GNM algorithm failed in such situations because log(1 + β_E_*X*) is not defined when (1 + β_E_*X*) ≤ 0, so we replaced such situations with a new run. Occasionally the GNM algorithm terminated successfully but with no convergence for the discrete-variable (parity) parameter; this occurred at most only a few times per 2,000 runs, most frequently with smaller sample sizes and lower outcome prevalence. Failure of the continuous-variable (BMI) parameter to converge was extremely rare, but did occur. Runs where either the BMI or parity parameter failed to converge were discarded and simulation summaries were calculated using only the complete results. No failures of convergence occurred with the logistic regression model.

**Figure 1.**
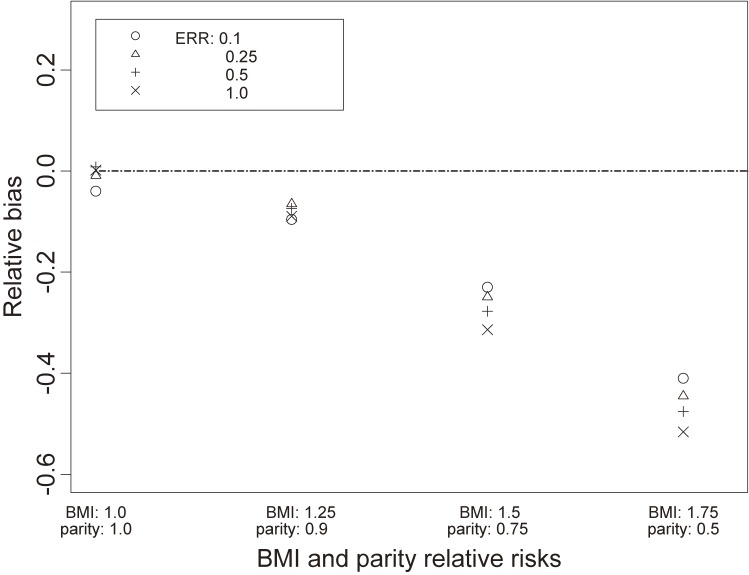
Simulated relative bias in the estimated excess relative risk (ERR) for exposure from a fit of model (2), as a function of true excess relative risk and various combinations of the relative risk (RR) of omitted covariates: body mass index (BMI) and parity. Cohort size *N* = 20,000, baseline prevalence *p*_0_ = 0.05, and control:case sampling ratio 2:1.

Simulated values of the estimated ERR ${\hat{\beta }}_{\text{E}}$ from a fit of model (2) demonstrated a slightly skewed distribution (see eMaterial 1). Therefore, we used the median of estimated ERR values over the 2,000 runs for estimating bias. Figure 1 shows the relative bias $[\text{median}({\hat{\beta }}_{\text{E}})-\beta_{\text{E}}]/\beta_{\text{E}}$ for various pairs of BMI and parity effects, ranging from small to large and for the four different values of true exposure ERR β_E_. The farther from null the effects of the omitted covariates, the greater was the relative bias (greater attenuation). Relative bias of the ERR estimate ${\hat{\beta }}_{\text{E}}$ was also greater with larger magnitude of true exposure ERR β_E_, and this became more apparent with effects of omitted covariates farther from null.

The effect of omitting the protective discrete covariate parity was not as dramatic as that of omitting the deleterious covariate BMI (see eFigure 1). To clarify whether this was due to a detrimental versus protective effect or due to a continuous versus discrete covariate, we conducted simulations where the effect of the continuous covariate BMI took on inverse ‘protective’ values 1.25^−1^, and 1.5^−1^ (Table 2; more-detailed results are shown in eTable 1). The magnitude of bias and gain in precision of the ERR estimate ${\hat{\beta }}_{\text{E}}$ were not symmetric: for the same degree of risk (eg, RR = 1.25 versus RR = 1.25^−1^ = 0.8), the degree of attenuation bias and precision gain was less when the omitted continuous covariate had a protective effect than when it had a detrimental effect. Thus, the asymmetry does not seem to be due to the distinction between discrete and continuous covariates. We also observed the same qualitative difference in detrimental versus protective covariates with ordinary logistic regression (Table 2 and eTable 2). This asymmetry does not seem to have been reported in previous studies of omitted-covariate bias with logistic regression.

**Table 2.** Simulated effect of omitting a continuous covariate with either detrimental or protective effect^a^

Relative risk for omitted covariate	With covariate omitted		With covariate included	
	
	Median	Std dev	Median	Std dev
*ERR model, ERR for radiation 0.5*				
1.5	0.375	0.109	0.509	0.145
1.25	0.458	0.141	0.495	0.158
1.0	0.504	0.171	0.504	0.171
0.8 (1.25^−1^)	0.478	0.141	0.495	0.153
0.667 (1.5^−1^)	0.418	0.121	0.499	0.148

*Logistic regression, relative risk for radiation 1.5 (log relative risk = 0.405)*				
1.5	0.315	0.071	0.403	0.087
1.25	0.381	0.084	0.407	0.091
1.0	0.401	0.096	0.401	0.096
0.8 (1.25^−1^)	0.391	0.087	0.408	.090
0.667 (1.5^−1^)	0.350	0.073	0.406	0.085

ERR, excess relative risk.

^a^Based on 2,000 simulations with cohort size *N* = 20,000, baseline prevalence *p*_0_ = 0.05, and 2 controls per case.

Results on precision of the ERR estimate ${\hat{\beta }}_{\text{E}}$ with omitted covariates are presented in the eFigure 2. Within each simulation scenario, the standard deviation of simulated ERR estimates over all runs was virtually identical to the average across runs of the estimated standard errors. The standard deviation decreased (precision increased) with increasing magnitude of the RRs of the omitted covariates (RRs increasingly far from null). The decrease in standard deviation (gain in precision) of the ERR estimate ${\hat{\beta }}_{\text{E}}$ with covariate omission was most pronounced at the largest values of true exposure ERR β_E_, which have larger variance. As the RR of omitted BMI increased, the standard deviation of the estimated ERR values approached its lower bound of zero, so further decrease due to greater RRs of omitted covariates was less pronounced.

**Figure 2.**
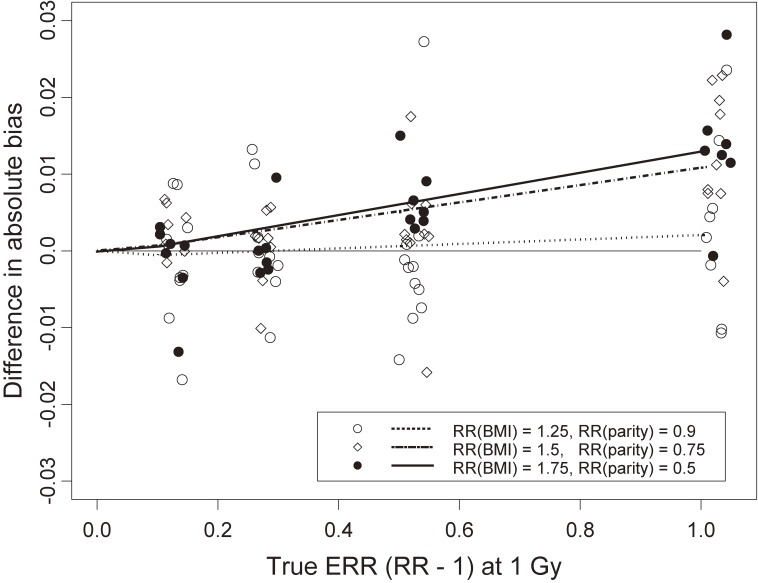
Simulated absolute bias: the difference between that for a true excess relative risk (ERR) model parameter (calculated with the generalized nonlinear model) and that for a true logistic regression model parameter (rescaled to ERR by [exp{log(RR)} − 1], where RR is relative risk), with risk at unit dose the same under each model. Includes all simulation configurations: cohort size *N* = 5,000 or 20,000; baseline prevalence *p*_0_ = 0.015, 0.05, or 0.1; and control:case ratio *m* = 2 or 5 (although some configurations could not be evaluated, as described in the text). Results for three different pairs of magnitudes of omitted-covariate effect sizes are shown (see figure legend). BMI, body mass index.

Figure 2 shows the difference in absolute attenuation bias between the true ERR model and the true logistic model, with data under each model generated using the same true RR for an exposure of one unit (ie, setting $[1+\beta_{\text{E}}]=e^{\beta_{\text{L}}}$). All values of cohort size, prevalence, and control:case ratio are included in Figure 2, as there was little effect of these parameters on relative bias (see eFigures 3, 4, and 5), although some configurations could not be evaluated (as explained previously). There was little difference in terms of bias between the ERR and logistic models at true RR values of 1.1 and 1.25. Bias with the ERR model increased compared to that with the logistic model at larger values of true RR, which is revealed by superimposing fitted curves obtained using the supsmu function, with span 0.4, in S-plus (version 6.2; Insightful Corp., Seattle, Washington). Bias with the ERR model also increased compared to that with the logistic model as RRs of omitted covariates became farther from null. Because the difference in biases of the two methods may be difficult to interpret, we also calculated the mean bias for each of the two methods and computed the ratio of those means at each true RR. The ratio of mean absolute bias in the estimated ERR parameter ${\hat{\beta }}_{\text{E}}$ to mean absolute bias in the log(RR) parameter ${\hat{\beta }}_{\text{L}}$ (on the ERR scale) was 1.017 at true RR = 1.5 and 1.034 at true RR = 2.0.

**Figure 3.**
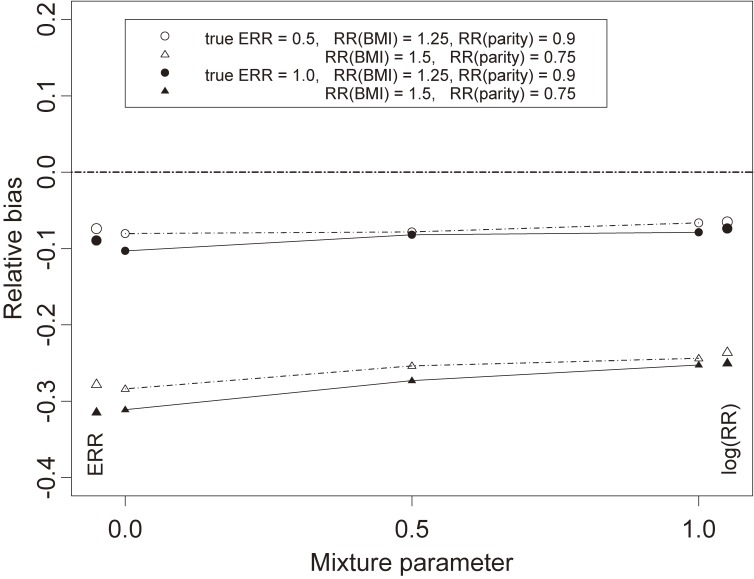
Simulated relative bias in the exposure risk estimate from the mixture model as a function of the mixture parameter. Based on cohort size *N* = 20,000, baseline prevalence *p*_0_ = 0.05, and control:case ratio *m* = 2. Simulated relative biases for the excess relative risk (ERR) and logistic regression (log RR, where RR is relative risk) models are shown next to their equivalents in the mixture model for comparison. BMI, body mass index.

Figure 3 shows how the bias of the parameter estimate ${\hat{\beta }}_{\text{M}}$ depends on the parameter λ in the mixture model (1). The magnitude of attenuation was greater at λ = 0 (ERR model) than at λ = 1 (logistic model), consistent with the slightly greater bias seen with the ERR model in Figure 2. Indeed, biases with the mixture model at λ = 0 and λ = 1 were respectively similar to those with the ERR and ordinary logistic regression models with the same values of RR (shown in the side margins of Figure 3). At λ = 0.5, the bias was intermediate to the biases at λ = 0 and λ = 1. Again, the relative bias was greater (more negative) with RRs of omitted covariates farther from null, and the relative bias was greater with larger values of true exposure RR.

## DISCUSSION

Contrary to conventional wisdom, omitting non-confounding risk factors from logistic regression models for binary outcomes is known to result in attenuation yet increased precision in the estimated effects of covariates included in the model; this is related to non-collapsibility of the odds ratio.^22^ We have shown that these results carry over to the ERR model and a more general relative-risk mixture model that includes the ERR model and ordinary logistic regression model as special cases, at least in the simple situation where there are no unmeasured or unadjusted confounders. Despite the further complications of a nonlinear link function and a nonlinear predictor, the bias with these general relative-risk models was similar to that with logistic regression for a true exposure RR up to 1.25 and only slightly larger with a true RR as high as 2.0. This is not surprising given that the ERR model can be approximated using a logistic model at small values of risk (see eMaterial 1, “**Logistic approximation to the ERR model**”). It seems to be the consensus of most authors that, with GLMs, it is better to adjust than not to adjust, particularly in moderate to large samples, because asymptotically the bias predominates over the variance.^1^ We conclude that the same is true with general relative-risk models for binary outcome data.

With logistic regression and with general relative-risk models, omitting a non-confounding predictive covariate can result in gain of precision. Thus, we have a dilemma in that omitting covariates can lead to a biased but more precise risk estimate. In practice, there may be some advantage to using the unadjusted risk estimate (with non-confounding predictive covariates omitted) for testing the null hypothesis of no risk. However, any advantage would apply only in small samples because with large samples the bias dominates the mean squared error.^1^ Furthermore, if the test is rejected, one is left with an attenuated risk estimate.

We focused on the ERR model because it provides a simple approach to modelling joint effects of multiple risk factors on an additive scale. The mixture model of Thomas^8^ is an intuitive method for testing additive versus multiplicative scales, and a similar model is available in the Epicure software package under the label ‘geometric mixture model’.^13^ However, when the best-fitting model is neither purely additive nor purely multiplicative, the same parameter appears in both the ERR and log-linear terms of the mixture model. Little et al^23^ noted that, “in the presence of ‘mixing’, one would not expect there to be common parameters in both”, but they reported having problems fitting separate risk parameters for the two exposure components of the mixture model. That difficulty was due in large part to the fact that one or the other risk parameter is not defined when the mixing parameter takes value 0 or 1. The power model might, therefore, be more intuitive for estimating risk when the fit of the mixture model suggests that the scale is neither the pure ERR nor the pure logistic.

We noted that omitting a covariate caused smaller bias when the covariate was simulated to have a protective effect with the same magnitude of RR but as the inverse. Moolgavkar and Venzon^24^ noted that both the mixture model and the power model suffer from the fact that recoding a binary covariate can affect the results, whereas a related model of Guerrero and Johnson,^25^ which differs slightly from the power model and is related to Box-Cox transformation, is not altered by recoding a binary covariate. We speculate that this may be related to the lack of symmetry between detrimental and protective covariates, which is analogous to switching reference and exposed groups with a dichotomous risk factor.

In conclusion, the effects—in terms of bias and precision—of omitting non-confounding predictor variables from general relative-risk models for binary data are similar both qualitatively and quantitatively to effects previously reported for logistic regression models, except with large values of risk. The best strategy seems to be to include covariates known or suspected to be risk factors, whether or not they are suspected of being confounders. Nevertheless, in practice it may be worthwhile to empirically assess the impact of omission before deciding whether to include a covariate. It would also be useful to assess in future research whether similar omitted-covariate-bias results hold when there are unmeasured confounding variables, as may often be the case in epidemiologic studies.

## APPENDIX A. SUPPLEMENTARY DATA

eMaterial 1. Effects of omitting non-confounding predictors from general relative-risk models for binary outcomes

eFigure 1. Simulated relative bias in the estimated ${\hat{\beta }}_{\text{E}}$ of exposure excess relative risk (ERR) as a function of omitted covariate effect sizes (BMI relative risk and parity relative risk separately) at true exposure ERR β_E_ = 0.5. Cohort size *N* = 20,000, baseline prevalence *p*_0_ = 0.05, and control:case sampling ratio 2:1.

eFigure 2. Simulation standard deviations of estimated exposure ERR ${\hat{\beta }}_{\text{E}}$. A: at radiation ERR = 0.5 with various combinations of effects of omitted covariates. B: at various values of radiation ERR using the same pairs of effects of omitted covariates as in Figure 1 (solid line for ERR = 0.5 in panel B corresponds to the results with the same symbols in panel A). Cohort size *N* = 20,000, baseline prevalence *p*_0_ = 0.05, control:case sampling ratio 2:1.

eFigure 3. Simulated relative bias (A) and simulation standard deviations (B) of estimated exposure ERR ${\hat{\beta }}_{\text{E}}$ by cohort size for various magnitudes of ERR (colors) and magnitudes of effects of the omitted covariates. Open circles are for BMI relative risk 1.25, parity relative risk 0.9; filled circles are for BMI relative risk 1.5, parity relative risk 0.75.

eFigure 4. Simulated relative bias (A) and simulation standard deviations (B) of estimated exposure ERR ${\hat{\beta }}_{\text{E}}$ by case prevalence for various magnitudes of ERR (colors) and magnitudes of effects of the omitted covariates. Open circles are for BMI relative risk 1.25, parity relative risk 0.9; filled circles are for BMI relative risk 1.5, parity relative risk 0.75.

eFigure 5. Simulated relative bias (A) and simulation standard deviations (B) of estimated exposure ERR ${\hat{\beta }}_{\text{E}}$ by case:control ratio for various magnitudes of ERR (colors) and magnitudes of effects of the omitted covariates. Open circles are for BMI relative risk 1.25, parity relative risk 0.9; filled circles are for BMI relative risk 1.5, parity relative risk 0.75.

eFigure 6. Distribution of estimated exposure ERR values (${\hat{\beta }}_{\text{E}}$) in the simulation with true ERR β_E_ = 0.25 when omitted covariates have no effect on outcome (cohort size 20,000, outcome prevalence 0.05, control:case ratio 5). The mean estimated ERR values ${\hat{\beta }}_{\text{E}}$ in this case was 0.259, whereas the median was 0.253, closer to the true value 0.25.

eTable 1. Results of omitting the continuous covariate when it has either detrimental or protective effect on outcome: Results for the ERR model (2,000 simulations)

eTable 2. Results of omitting the continuous covariate when it has either detrimental or protective effect on outcome: Results for logistic regression (2,000 simulations)
