# Supplementary material for: Effects of Omitting Non-confounding Predictors From General Relative-Risk Models for Binary Outcomes
Source: J Epidemiol. 2019 Mar 5;29(3):116–22. doi: 10.2188/jea.JE20170226 (PMC6375815; doi:10.2188/jea.JE20170226)
Supplement: Supplementary file 1 [file je-29-116-s001.pdf]

eMaterial 1. Effects of omitting non-confounding predictors from general relative-risk models for binary outcomes

R code for the generalized nonlinear models

The excess relative risk (ERR) model fit is performed using the call

```
gnm(caseind ~ LogERR(dose) + bmi + parity, family=binomial,
  data=cc.data)
```

with the nonlinear function LogERR defined by

```
LogERR <- function(expression, inst = NULL){
    list(predictors = list(substitute(expression)),
         term = function(predictors, ...) {
            paste("log(1+", predictors, ")", sep = "")
         },
         call = as.expression(match.call()))
}
class(LogERR) <- "nonlin"
```

The mixture model is fit with the call

```
gnm(caseind ~ MixMod(dose,lambda) + bmi + parity, family=binomial,
data=cc.data)
```

where the nonlinear function MixMod is defined by

```
MixMod <- function(expression, lam, inst = NULL){
    list( predictors = list(substitute(expression)),
         term = function(predictors, ...) {
            paste(as.character(lam),"*", predictors, "+ (1-",
             as.character(lam), ")*log(1+", predictors, ")", sep
             = "")
         },
         call = as.expression(match.call()))
}
```

```
class(MixMod) <- "nonlin"
```

Summary data from each of the simulation runs (in a comma separated text file), as well as the simulation R script and the Splus functions used to summarize the results, can be provided upon request.

Logistic approximation to the ERR model

Here we note some algebraic connections between the ERR model and logistic regression. The ERR model can be written $\text{logit}(p) = \boldsymbol{\alpha}'\boldsymbol{Z} + \ln\left(1+\beta_{\text{E}}X\right)$ (equation (3) of the main paper). By Taylor series approximation, $\ln(1+\beta X) \approx \beta X$ when βX is small, because $\ln(1+\beta X) = \sum_{n=1}^{\infty}(-1)^{n+1}(\beta X)^n/\text{n}$ for $-1 < \beta X \leq 1$. We can also obtain this result by using the approximation $e^{\beta X} = 1+\beta X + O\left([\beta X]^2\right)$ for small βX. Thus, to an approximation depending on the size of βX, $\text{logit}(p) \approx \boldsymbol{\alpha}'\boldsymbol{Z} + \beta_{\text{E}}X$. In other words, the ERR model can be approximated by a logistic model if $\beta_{\text{E}}X$ is not too large, so we would expect that the results concerning bias and precision with omitted covariates in logistic regression should apply approximately to ~~the~~ a possibly mis-specified ERR $\hat{\beta}_{\text{E}}^{*}$ estimated with omitted covariates (equation (4) of the main paper). That means, for relatively small risk (effect) or small dose (because βX depends on both), omitting a risk factor Z not associated with X should lead to attenuation bias but greater precision in the estimated ERR, with the degree of bias and degree of gain in precision positively related to the magnitude of effect of the omitted covariate (how far that effect is from null). However, these similarities are not exact, and indeed the approximation would break down with values of risk far from zero.

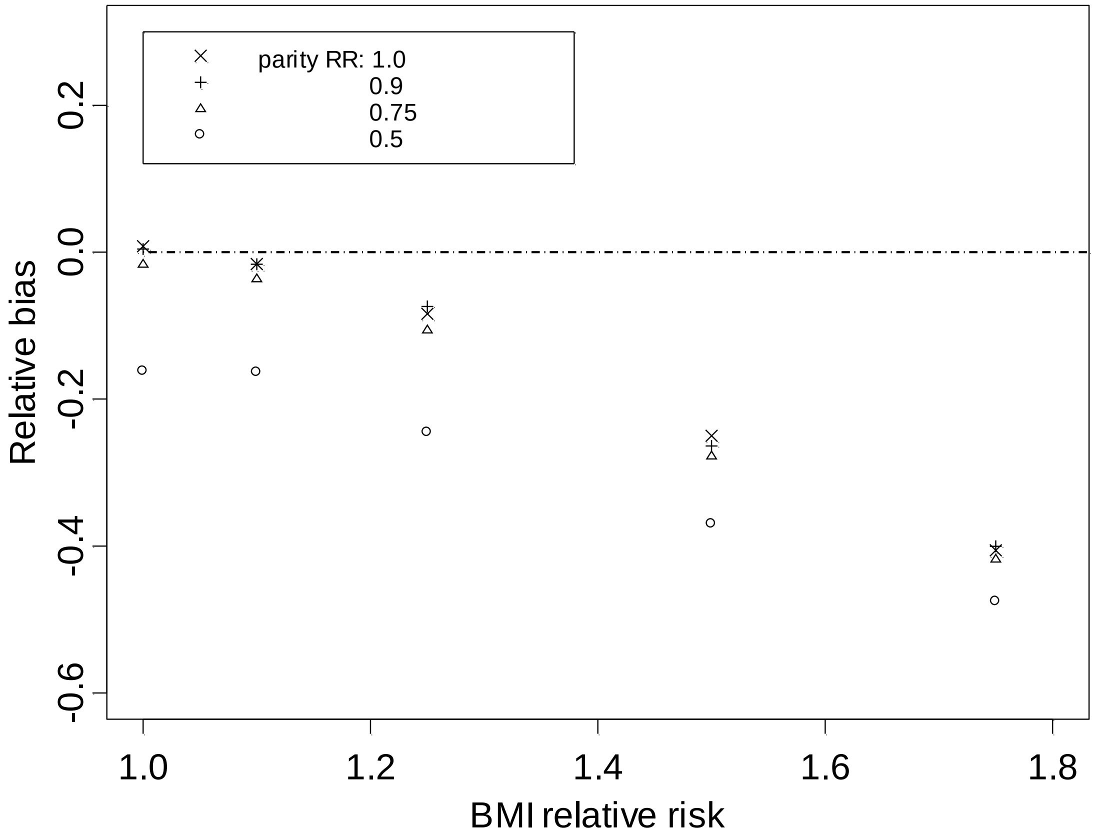

eFigure 1. Simulated relative bias in the estimated $\hat{\beta}_{\mathrm{E}}$ of exposure excess relative risk (ERR) as a function of omitted covariate effect sizes (BMI relative risk and parity relative risk separately) at true exposure ERR $\beta_{\mathrm{E}} = 0.5$. Cohort size N = 20,000, baseline prevalence p_0 = 0.05, and control:case sampling ratio 2:1.

eTable 1. Results of omitting the continuous covariate when it has either detrimental or protective effect on outcome: Results for the ERR model (2,000 simulations)

Relative risk of omitted covariate BMI	With covariate omitted		With covariate included	
	Median ERR $\hat{\beta}_E$	SD	Median ERR $\hat{\beta}_E$	SD
True ERR for radiation $\beta_E = 0.25$				
1.5	0.194	0.097	0.252	0.126
1.25	0.234	0.119	0.252	0.130
1.0	0.248	0.137	0.249	0.137
0.8 (1.25^{-1})	0.242	0.126	0.255	0.133
0.667 (1.5^{-1})	0.212	0.102	0.247	0.120
True ERR for radiation $\beta_E = 1.0$				
1.5	0.706	0.135	0.994	0.195
1.25	0.907	0.181	1.000	0.206
1.0	1.002	0.212	1.003	0.212
0.8 (1.25^{-1})	0.944	0.184	1.007	0.202
0.667 (1.5^{-1})	0.803	0.147	0.999	0.192

Based on cohort size N = 20,000, baseline prevalence p_0 = 0.05, and 2 controls per case. Relative risk for the discrete covariate, parity, was 1.0, so that it did not affect the bias/precision (i.e. results were the same whether or not parity was omitted).

Abbreviations: BMI: body mass index; ERR, excess relative risk; SD, standard deviation.

eTable 2. Results of omitting the continuous covariate when it has either detrimental or protective effect on outcome: Results for logistic regression (2,000 simulations)

Relative risk of omitted covariate BMI	With covariate omitted		With covariate included	
	Median of $\hat{\beta}_L$	SD	Median of $\hat{\beta}_L$	SD
True RR for radiation 1.25 (log RR β_L = 0.223)				
1.5	0.176	0.075	0.226	0.091
1.25	0.212	0.090	0.226	0.096
1.0	0.223	0.103	0.223	0.103
0.8 (1.25^{-1})	0.218	0.095	0.224	0.100
0.667 (1.5^{-1})	0.198	0.078	0.222	0.091
True RR for radiation 2.0 (log RR β_L = 0.693)				
1.5	0.542	0.068	0.697	0.080
1.25	0.648	0.079	0.692	0.084
1.0	0.694	0.089	0.694	0.089
0.8 (1.25^{-1})	0.662	0.081	0.692	0.084
0.667 (1.5^{-1})	0.586	0.068	0.692	0.079

BMI, body mass index; RR, relative risk; SD, standard deviation.

Based on cohort size N = 20,000, baseline prevalence p_0 = 0.05, and 2 controls per case. Relative risk for the discrete covariate, parity, was 1.0, so that it did not affect the bias/precision (i.e. results were the same whether or not parity was omitted).

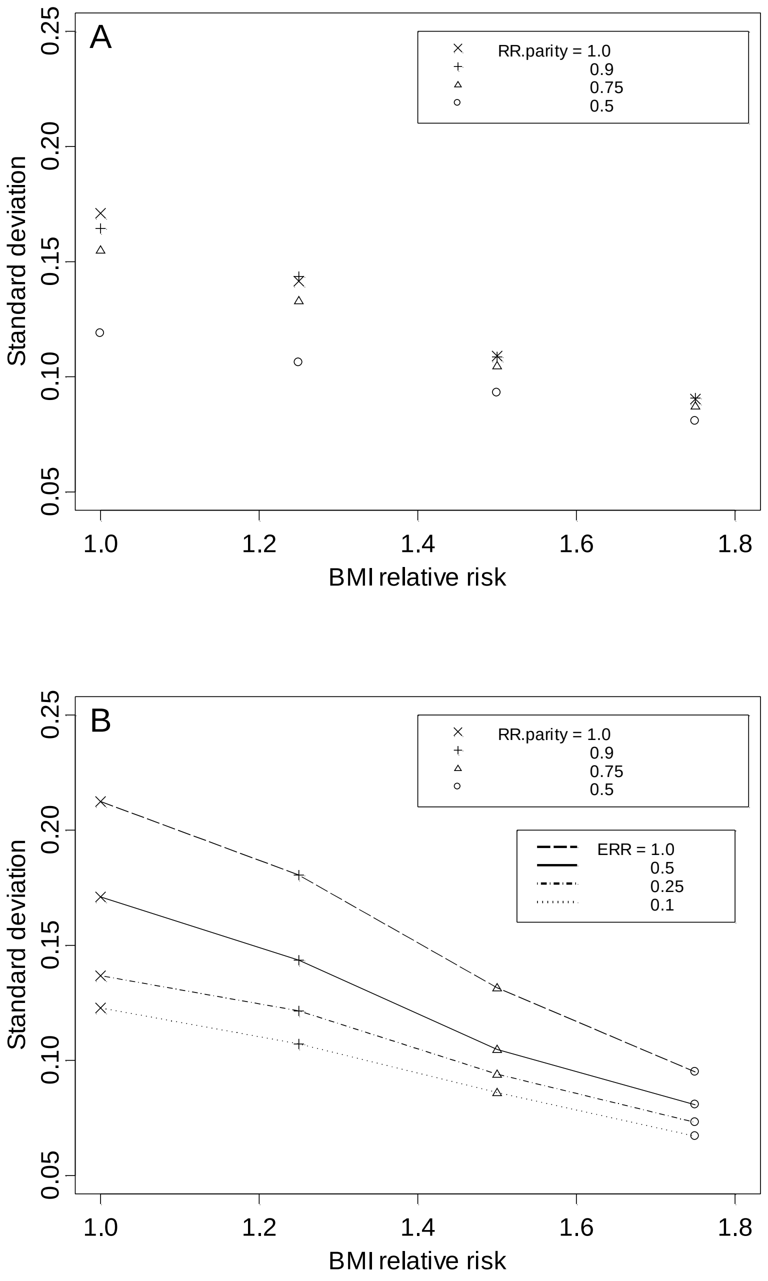

eFigure 2. Simulation standard deviations of estimated exposure ERR $\hat{\beta}_E$. A: at radiation ERR=0.5 with various combinations of effects of omitted covariates. B: at various values of radiation ERR using the same pairs of effects of omitted covariates as in Figure 1 (solid line for ERR=0.5 in panel B corresponds to the results with the same symbols in panel A). Cohort size N=20,000, baseline prevalence $p_0 = 0.05$, control:case sampling ratio 2:1.

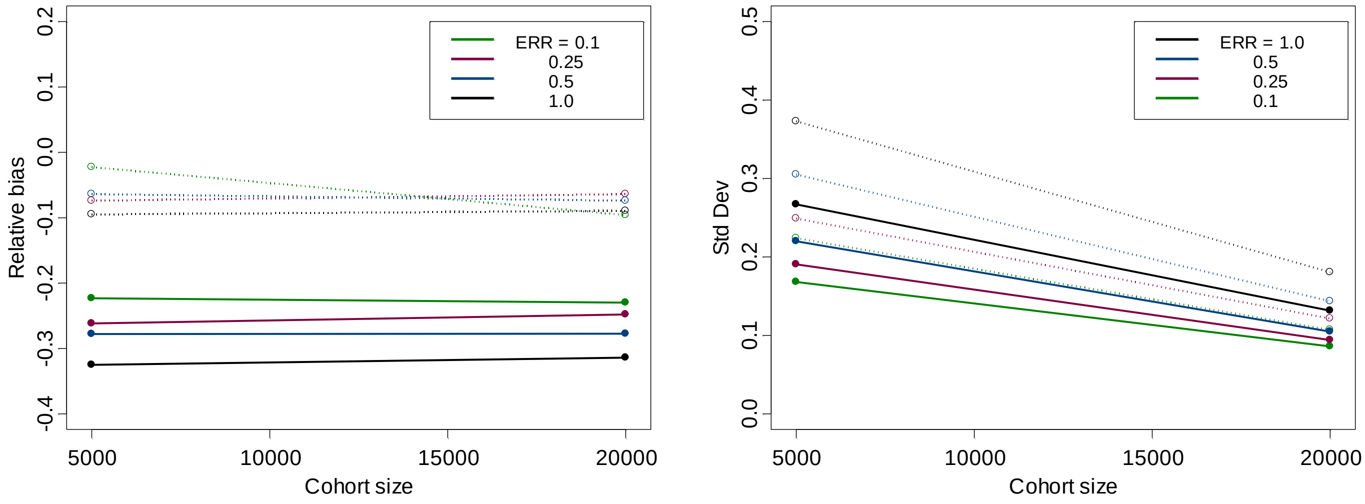

eFigure 3. Simulated relative bias (A) and simulation standard deviations (B) of estimated exposure ERR $\hat{\beta}_{\mathrm{E}}$ by cohort size for various magnitudes of ERR (colors) and magnitudes of effects of the omitted covariates. Open circles are for BMI relative risk 1.25, parity relative risk 0.9; filled circles are for BMI relative risk 1.5, parity relative risk 0.75.

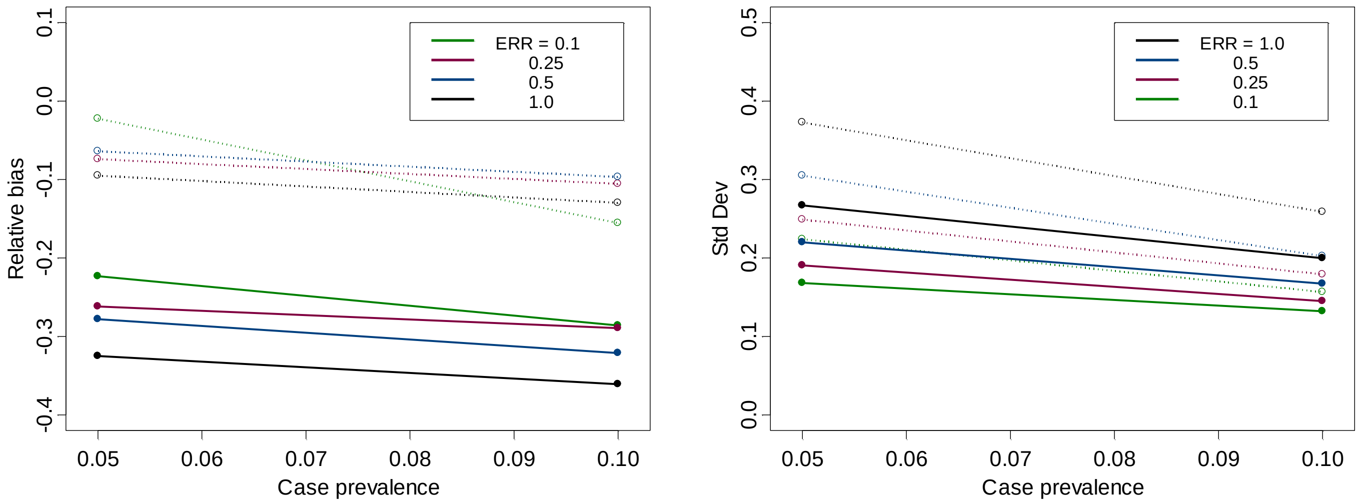

eFigure 4. Simulated relative bias (A) and simulation standard deviations (B) of estimated exposure ERR $\hat{\beta}_{\mathrm{E}}$ by case prevalence for various magnitudes of ERR (colors) and magnitudes of effects of the omitted covariates. Open circles are for BMI relative risk 1.25, parity relative risk 0.9; filled circles are for BMI relative risk 1.5, parity relative risk 0.75.

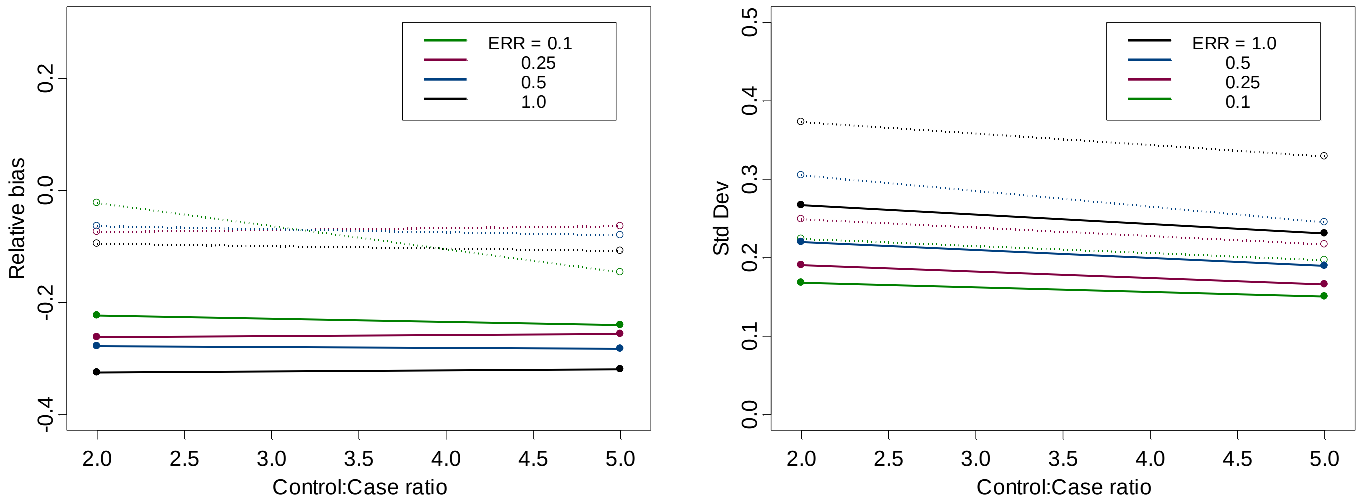

eFigure 5. Simulated relative bias (A) and simulation standard deviations (B) of estimated exposure ERR $\hat{\beta}_E$ by case:control ratio for various magnitudes of ERR (colors) and magnitudes of effects of the omitted covariates. Open circles are for BMI relative risk 1.25, parity relative risk 0.9; filled circles are for BMI relative risk 1.5, parity relative risk 0.75.

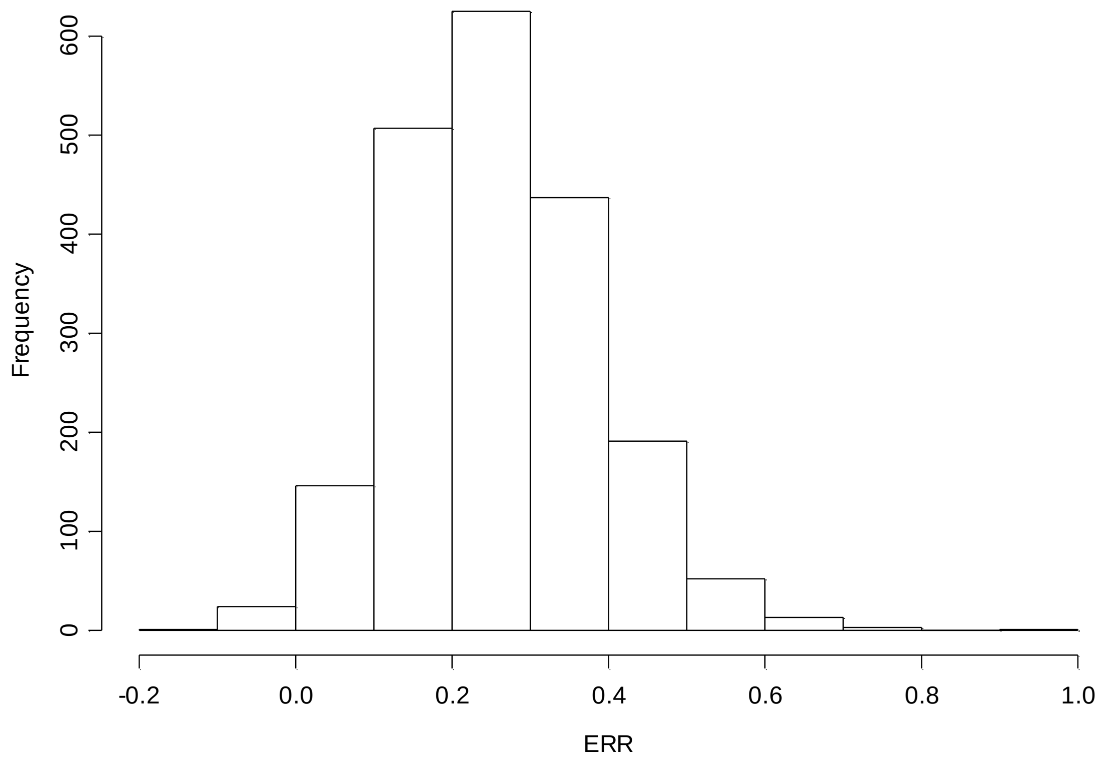

eFigure 6. Distribution of estimated exposure ERR values ($\hat{\beta}_E$) in the simulation with true ERR $\beta_E = 0.25$ when omitted covariates have no effect on outcome (cohort size 20,000, outcome prevalence 0.05, control:case ratio 5). The mean estimated ERR values $\hat{\beta}_E$ in this case was 0.259, whereas the median was 0.253, closer to the true value 0.25.
